# Electroencephalographic Changes Associated with Antinociceptive Actions of Lidocaine, Ketamine, Meloxicam, and Morphine Administration in Minimally Anaesthetized Dogs

**DOI:** 10.1155/2015/305367

**Published:** 2015-01-28

**Authors:** Ubedullah Kaka, Chen Hui Cheng, Goh Yong Meng, Sharida Fakurazi, Asmatullah Kaka, Atique Ahmed Behan, Mahdi Ebrahimi

**Affiliations:** ^1^Department of Veterinary Clinical Studies, Faculty of Veterinary Medicine, Universiti Putra Malaysia, 43400 Serdang, Selangor, Malaysia; ^2^Faculty of Animal Husbandry & Veterinary Sciences, Sindh Agriculture University Tandojam, Sindh 70060, Pakistan; ^3^Department of Veterinary Preclinical Sciences, Faculty of Veterinary Medicine, Universiti Putra Malaysia, 43400 Serdang, Selangor, Malaysia; ^4^Institutes of Tropical Agriculture, Universiti Putra Malaysia, 43400 Serdang, Selangor, Malaysia; ^5^Laboratory of Vaccines and Immunotherapeutics, Institute of Bioscience, Universiti Putra Malaysia, 43400 Serdang, Selangor, Malaysia; ^6^Department of Human Anatomy, Faculty of Medicine and Health Science, Universiti Putra Malaysia, 43400 Serdang, Selangor, Malaysia; ^7^Department of Animal Sciences, Faculty of Agriculture, Universiti Putra Malaysia, 43400 Serdang, Selangor, Malaysia

## Abstract

Effects of ketamine and lidocaine on electroencephalographic (EEG) changes were evaluated in minimally anaesthetized dogs, subjected to electric stimulus. Six dogs were subjected to six treatments in a crossover design with a washout period of one week. Dogs were subjected to intravenous boluses of lidocaine 2 mg/kg, ketamine 3 mg/kg, meloxicam 0.2 mg/kg, morphine 0.2 mg/kg and loading doses of lidocaine 2 mg/kg followed by continuous rate infusion (CRI) of 50 and 100 mcg/kg/min, and ketamine 3 mg/kg followed by CRI of 10 and 50 mcg/kg/min. Electroencephalogram was recorded during electrical stimulation prior to any drug treatment (before treatment) and during electrical stimulation following treatment with the drugs (after treatment) under anaesthesia. Anaesthesia was induced with propofol and maintained with halothane at a stable concentration between 0.85 and 0.95%. Pretreatment median frequency was evidently increased (*P* < 0.05) for all treatment groups. Lidocaine, ketamine, and morphine depressed the median frequency resulting from the posttreatment stimulation. The depression of median frequency suggested evident antinociceptive effects of these treatments in dogs. It is therefore concluded that lidocaine and ketamine can be used in the analgesic protocol for the postoperative pain management in dogs.

## 1. Introduction

Lidocaine and ketamine are widely used in the veterinary practice. Lidocaine has been used as local anesthetic and antiarrhythmic agent, whereas ketamine has been used as a short acting general anesthetic. The trend of using lidocaine and ketamine for postoperative analgesia in veterinary practice is gaining acceptance with increasing evidences supporting their beneficial effects. The major benefit of lidocaine and ketamine use for postoperative analgesia may be preventing the development of central sensitization [1] during surgical intervention, which augments pain and discomfort in the postoperative period. Lidocaine and ketamine have been reported to supplement general anaesthesia [2] reducing the amount of inhalant anaesthetics required during anaesthesia, improving cardiorespiratory function, and thereby provide safe general anaesthesia, better postoperative comfort, and quicker recovery.

The combination of lidocaine and ketamine as a nonopioid adjunct can enhance efficacy, potential for drug synergism, and decrease drug-related side effects [3] and reduce the opioid requirement and their side effects in postoperative period [4]. Intravenous lidocaine has also been reported to produce gastrointestinal promotility and antishock effects and decrease amount of injectable or inhalant anaesthetics [5]. Minimum alveolar concentration (MAC) of isoflurane and sevoflurane has been reported to be reduced when used in conjunction with continuous rate infusion (CRI) of lidocaine [6, 7] and ketamine [8, 9] in dogs. These suggested that the usage of lidocaine and ketamine may have attenuated the intensity of nociceptive signals being transmitted to the central nervous system, thus lowering the requirement for general anesthetics. In fact, ketamine and lidocaine combination was also shown to reduce the MAC of sevoflurane by 62.8% in dogs [10] and 69.4% in goats [11]. Therefore, the combined use of lidocaine and ketamine with gas anaesthetics would potentially lead to a safer anaesthetic regime, while addressing pain and nociception associated with invasive procedures during anaesthesia.

The electroencephalogram (EEG) is the recording of electrical activity from electrodes placed in various positions on the scalp in human and head in other species [12]. Electroencephalogram spectrum changes have been used as a tool to evaluate nociceptive response in ponies [13], in red deer [14], in dogs [15, 16], in horses [13], in sheep [17], in pigs [18], and in cattle [19, 20]. Under the “minimal anaesthesia model,” [12] animals have been shown to be able to demonstrate EEG responses from the cerebral cortex as well as normal physiological cardiovascular functions to nociceptive stimulation that are consistent to fully awake animals without experiencing pain [15, 18]. This model has been used and reported in cattle [20], deer [14], wallabies [21], horses [13], sheep [17], and dogs [15]. The minimal anaesthesia model uses the EEG response to noxious electrical stimulation as a tool to evaluate the efficacy of centrally acting agents [22]. Noxious stimulation elicits transmission of nociceptive action potentials under general anaesthesia [23]. It activates medullary centers of circulation and ventilation, hypothalamic centers of neuroendocrine function, and limbic structures. This is exhibited as increased sympathetic tone, systemic vascular resistance, stroke volume, heart rate, cardiac output, arterial pressure, metabolic rate, and oxygen consumption as well as hyperventilation. Endocrine responses are associated with the increase in adrenocorticotropic hormone, catecholamine, cortisol, antidiuretic, and growth hormone with parallel decrease in insulin and testosterone. This is further accompanied by changes in electrolyte and metabolic responses [24]. Commonly used potent analgesic agents have been reported to prevent or attenuate changes in EEG variables of nociception [25] and reduce minimum alveolar concentration (MAC) in dogs [26].

Systemic lidocaine [27, 28] and ketamine [29, 30] are known to have analgesic effects in humans, even though lidocaine is being used traditionally as an antiarrhythmic drug when delivered systematically. The literature regarding systemic antinociceptive effects of lidocaine and ketamine in dogs is scarce and available studies did not provide conclusive evidence on their antinociceptive effects when used as a systemic drug. A number of reports concluded that lidocaine did not have evident analgesic effects [31, 32]; however, there are some reports [33–35] that contradicted this. On a similar note, some researchers noted that ketamine did demonstrate good analgesic effects when administered systematically [36, 37], while a minority number of reports [38] suggested otherwise. In fact, the data on effective analgesic concentration of ketamine is not available at all [39]. Dosages used in earlier animal studies [36–38] were based on human studies and were very different from each other. All these inconsistencies were attributed to the fact that nociceptive assessments in these reports were mostly based on behavioral scales. Most of these scales have not been validated for reliability, specificity, or linearity, and crucially most of the scales are based on subjective assessment of behaviour [40, 41]. In contrast, methods based on electroencephalography are able to not only record instantaneous response to nociception but represent an empirical approach to assess nociception in animal subjects [19]. In fact, electroencephalography has been used as a tool for objective measurement of analgesic effects of drugs in question [13]. Therefore, this study attempted to investigate the antinociceptive effects of lidocaine and ketamine administered systemically in response to electric stimulation in dogs anaesthetized with halothane using electroencephalography. It was hypothesized that systemic lidocaine and ketamine at subanaesthetic dosage are antinociceptive and therefore would depress changes in EEG spectrum in response to noxious stimulation in the dog model.

## 2. Materials and Methods

### 2.1. Experimental Design

The study was subjected to the review and approved by the Universiti Putra Malaysia Animal Care and Utility Committee (UPM/IACUC/AUP-R023/2013). Six healthy adult mixed-breed female dogs weighing 15.17 ± 1.94 (mean ± SD) were used in this study. Dogs were judged healthy based on physical examination, hematology, and blood biochemistry. Following one-month acclimatization, dogs were subjected to six treatment protocols in a crossover Latin square design. Wash-out period was 7 days between treatments. The six treatment protocols were (1) lidocaine 2 mg/kg, IV bolus (LLD), (2) ketamine 3 mg/kg, IV bolus (KLD), (3) meloxicam 0.2 mg/kg, IV bolus (MLX), (4) morphine 0.2 mg/kg, IV bolus (MRP), (5) lidocaine 2 mg/kg, IV bolus, followed by CRI of 50 mcg/kg/min and 100 mcg/kg/min lidocaine (LCRI), and (6) ketamine 3 mg/kg, IV bolus, followed by CRI of 10 mcg/kg/min and 50 mcg/kg (KCRI).

### 2.2. Anaesthesia Protocol

Animals were fasted for 12 hours prior to anaesthesia with free access to water. Catheter (20 SWG) was placed in left/right cephalic vein. Anaesthesia was induced with propofol 5 mg/kg and maintained with halothane in 100% oxygen. Vaporizer was adjusted to maintain end-tidal halothane tension (*E*_THAL_) between 0.85% and 0.95%. All animals breathed spontaneously. Animals were positioned on right lateral recumbency. Lactated Ringer's solution was administered to maintain mean blood pressure above 60 mm Hg throughout the anesthetic period. A blood pressure cuff of 40–60% circumference of the antebrachium was used to measure blood pressures. All parameters were monitored using Datex-Ohmeda monitor (GE healthcare, Finland Oy, Helsinki, Finland). The temperature was maintained between 37 and 38°C using a heating pad and warm blanket.

### 2.3. Experimental Procedure

Following induction, dogs were maintained on halothane for 90 minutes to allow instrumentation and minimize residual effect of propofol. Baseline EEG data were recorded for 10 minutes, before (T0b) and after (T0a) electric stimulation. Ten minutes after the baseline stimulation, drugs were administered. For LLD, KLD, MLX, and MRP, the EEG data before and after electric stimulation were collected at 5, 20, and 40 minutes after drug administration. For LCRI and KCRI, the loading doses were administered, followed by the lower CRI doses for 20 minutes. The EEG data before and after the electric stimulation were collected. Then, the higher CRI doses were administered for another 20 minutes, and the EEG data collection before and after electrical stimulation was repeated. Noxious electrical stimulus was delivered with a peripheral nerve stimulator N272 (Fisher and Paykel Healthcare international, New Zealand) at 40 mA [42] and 50 Hz for 5 seconds. As per user manual, the device can provide maximum output voltage of 350 ± 10% V in external mode, which was the mode employed for this study. The stimulus was applied to the left hind limb (lateral aspect of the distal metatarsus) through two subdermal needle electrodes placed subcutaneously 2 cm apart. At the end of each experiment, halothane was disconnected and dogs were extubated when the laryngeal reflexes returned.

### 2.4. ECG, EEG, and Electric Stimulation

The electroencephalogram was recorded using a personal computer installed with Chart 5.5.5 recording software and connected to Powerlab 4/20 data recording system (Powerlab data acquisition system, AD Instruments Ltd. Sydney, Australia). Three stainless steel sterile disposable acupuncture needles (Wuxi Jiajian Medical Instrument Co., Ltd. Wuxi, Jiangsu, China) were placed subcutaneously, with the inverting electrode over the zygomatic process of left frontal bone, the noninverting electrode over the left mastoid process, and the ground electrode caudal to the occipital process [12]. Care was taken to ensure that the total impedance of the circuitry was less than 5 kOhms.

The electroencephalogram was recorded at a sampling rate of 1 kHz and raw EEG was resampled with low pass filter of 200 Hz into delta frequency (0.1 to 4 Hz), theta frequency (4.1 to 8 Hz), alpha frequency (8.1 to 12 Hz), and beta frequency (12.1 to 20 Hz) as reported earlier [19]. Electroencephalogram data were collected for 10 min after electrical stimulation. Electrocardiogram was recorded continuously in the standard lead II configuration, with the negative electrode on the right forelimb and positive electrode on the left hind limb.

Analysis of the EEG data was performed offline after the completion of experiments. The median frequency (MF) and total EEG power (Ptot) were calculated for consecutive nonoverlapping 1-second epochs. Power density data were derived using a Cosine-Bell function. Electrical and mechanical interference were excluded from EEG data during stimulus application by excluding wave signals five to seven seconds before and after the nociceptive electrical stimulus. EEG data from 10-second blocks before to 10-second blocks after the electrical stimulus (after excluding five to seven second blocks immediately before and after the stimulus) were taken for statistical analysis [19].

Heart rate was derived manually from the ECG data. Prestimulation mean heart rate (PRE300) for each animal was calculated for a period of 300 seconds prior to stimulation. Poststimulation mean heart rate was expressed as the percentage of PRE300 values and calculated at intervals of 15 seconds (after 15, after 30 up to after 300) until 5 minutes after stimulus. Mean heart rate for the immediate 15 seconds before stimulation (before 15) was calculated and expressed as percent of 300.

### 2.5. Statistical Analysis

The results are presented as means ± standard error of the mean. Statistical analysis was performed by using the SAS software package, version 9.1 (SAS Inst. Inc., Cary, NC). The datasets were compared across treatment groups and time of measurement using the ANOVA procedure. Significantly different means were then elucidated using the Duncan Multiple Range Test (DMRT). All procedures were conducted at 95% confidence level.

## 3. Results

It is apparent that ketamine CRI, lidocaine CRI, and morphine depressed the MF after posttreatment stimulation compared to the pretreatment stimulation. However, MLX did not depress the posttreatment MF. There was no significant difference in the MF at pre- and posttreatment stimulation after MLX treatment. Absolute median frequency values increased significantly (*P* < 0.05) after electrical stimulation compared to baseline in all treatment groups. This was equivalent to a change of 105% and 119%, for KCRI and LCRI (Table 1), and 108%, 112%, 173%, and 124% for KLD, LLD, MRP, and MLX, respectively, from their initial MF baseline values (Table 2). Ketamine CRI at 10 and 50 mcg/kg/min and LCRI at 50 and 100 mcg/kg/min prevented the increase in MF significantly compared to pretreatment stimulation. There was no significant difference (*P* > 0.05) between the effects of 10 and 50 mcg/kg/min in KCRI and 50 and 100 mcg/kg/min in LCRI treatments (Table 1).

Ketamine and lidocaine bolus significantly (*P* < 0.05) depressed MF at 5 minutes posttreatment stimulus compared to pretreatment stimulus whereas they failed to prevent the rise in MF at 20 and 40 minutes. Morphine significantly (*P* < 0.05) prevented increases in MF at 5, 20, and 40 minutes posttreatment stimulation compared to pretreatment stimulation. Meloxicam failed to prevent change in MF at all stimulations. There was no difference in MF values between pre- and posttreatment stimulations in MLX group (Table 2).

The absolute total power of the EEG values decreased significantly (*P* < 0.05) after electrical stimulation in all treatment groups, except in LLD and LCRI groups. This was equivalent to a change of about 14% and 10% in KCRI and LCRI (Table 3), and 18%, 20%, 7%, and 17% for KLD, LLD, MRP, and MLX, respectively, from their initial Ptot baseline values (Table 4). Posttreatment Ptot also decreased significantly (*P* < 0.05) in all treatment groups except MRP at 5, 20, and 40 minutes and KLD at 5 minutes posttreatment stimulation. Total power also did not change at 20 and 40 minutes posttreatment stimulation in LLD group.

**Figure 1:**
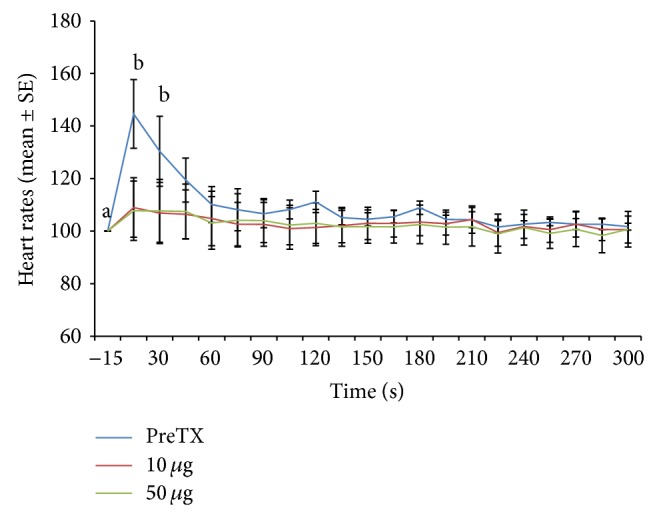
Mean ± SE for pre- and posttreatment heart rates expressed as percent of prestimulation rate of 6 dogs treated with two doses of ketamine CRI. Means with different superscripts are significantly different at (*P* < 0.05) within same time points.

**Figure 2:**
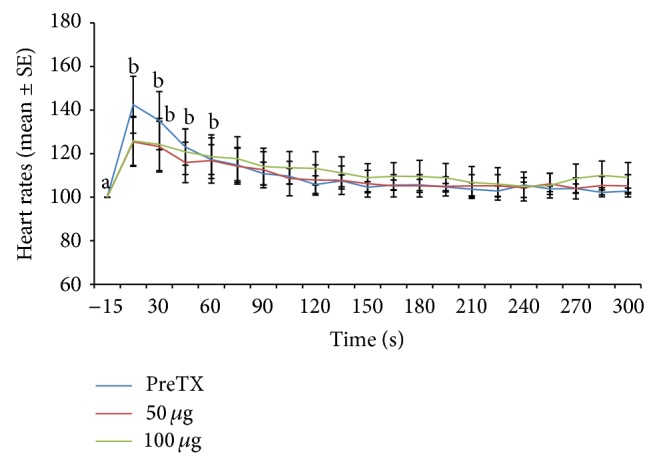
Mean ± SE for pre- and posttreatment heart rates expressed as percent of prestimulation rate of 6 dogs treated with two doses of lidocaine CRI. Means with different superscripts are significantly different from pre-15 value (*P* < 0.05) within same time point.

**Figure 3:**
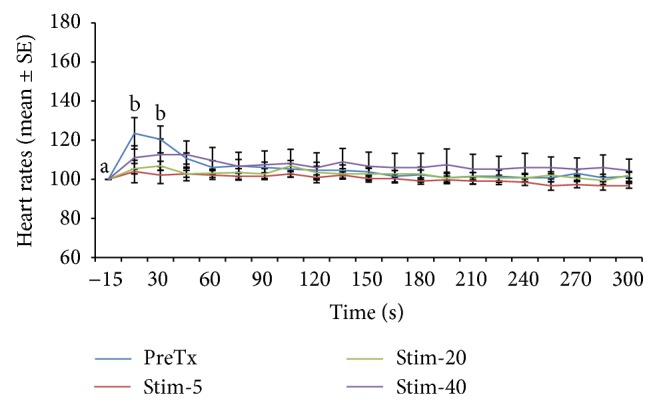
Mean ± SE for pretreatment, 5, 20, and 40 minutes posttreatment heart rates expressed as percent of prestimulation rate of 6 dogs treated with ketamine bolus. Means with different superscripts are significantly different at (*P* < 0.05) within same time point.

**Figure 4:**
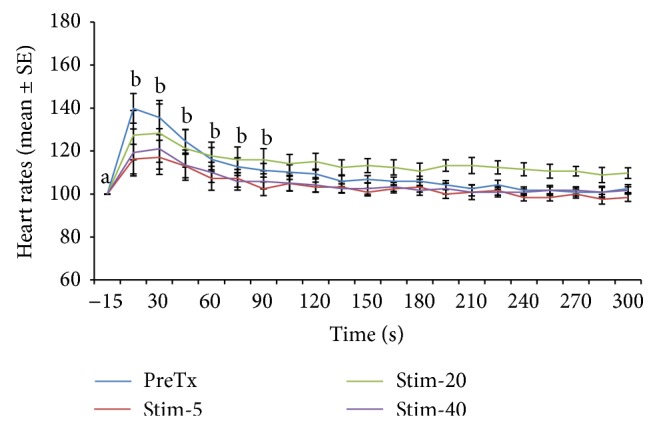
Mean ± SE for pretreatment and 5, 20, and 40 minutes posttreatment heart rates expressed as percent of prestimulation rate of 6 dogs treated with lidocaine bolus. Means with different superscripts are significantly different at (*P* < 0.05) within same time point.

**Figure 5:**
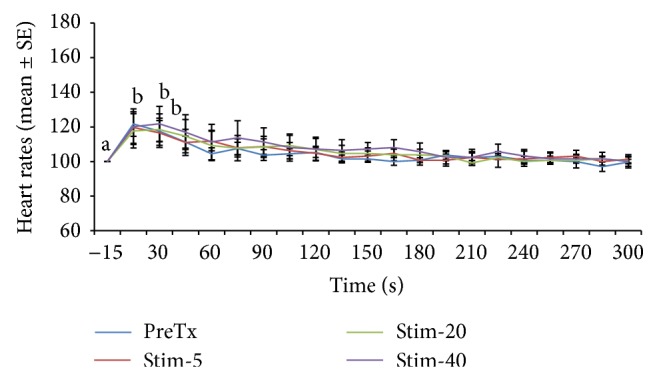
Mean ± SE for pretreatment, 5, 20, and 40 minutes posttreatment heart rates expressed as percent of prestimulation rate of 6 dogs treated with meloxicam bolus. Means with different superscripts are significantly different at (*P* < 0.05) within same time point.

**Figure 6:**
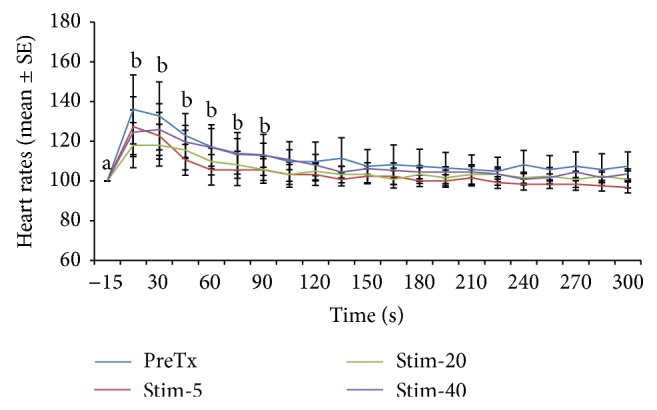
Mean ± SE for pretreatment and 5, 20, and 40 minutes posttreatment heart rates expressed as percent of prestimulation rate of 6 dogs treated with morphine bolus. Means with different superscripts are significantly different at (*P* < 0.05) within same time point.

Heart rate changes expressed as percentage of the prestimulation rate are depicted in Figures 1, 2, 3, 4, 5, and 6. In general, electrical stimulus applied prior to the administration of any of the drugs resulted in evident spiking of the heart rate for all treatment groups. The heart rates fell and normalize gradually within 45–60 seconds after the stimulus. In cases where ketamine (Figure 1) and lidocaine (Figure 2) were given as continuous rate infusion (CRI), the poststimulus spiking of the heart rate was barely noted. Furthermore, the CRI dosages for both ketamine and lidocaine were not related to the degree of suppression of the heart rate. However, the poststimulus dose-related suppression of heart rate becomes evident when ketamine (Figure 3) and lidocaine (Figure 4) were delivered as boluses. On the contrary, both meloxicam and morphine demonstrated negligible effects on the poststimulus heart rate.

## 4. Discussion

Most of the parameters showed attenuated response following ketamine, lidocaine, and morphine administration. Lidocaine and ketamine showed the most apparent depression, particularly when given as CRI. Lidocaine and ketamine administered as CRI significantly depressed the increase in MF in response to electrical stimulation. There was a significant difference in the MF between pre- and posttreatment stimulation. This is similar to the findings in ponies given an intravenous infusion of lidocaine before castration that demonstrated the depression of MF [13]. However, ketamine had negligible effects on the MF in horses under halothane anaesthesia [43]. Other studies have also shown a decrease in the MF in response to analgesic agents such as alfentanil [25]. The doses of ketamine and lidocaine used in this study were based on the doses used in MAC studies [2, 10] as well as used in the awake dogs [32, 39]. Effective analgesic plasma concentration of ketamine in humans ranges from 100 to 200 ng/mL [44]. Extrapolating from human surgical studies, Bergadano et al. [39] used ketamine at the dose of 0.5 mg/kg bolus followed by 10 mcg/kg/min CRI and studied the effects of low dose ketamine on repeated nociceptive stimuli in conscious dogs [39]. They found that plasma concentration during CRI was 5 times lower than that detected in human plasma concentration. We used 3 mg/kg bolus followed by 10 and 50 mcg/kg ketamine, a dose 5 times higher than that used by Bergadano et al. [39] in conscious dogs. Therefore, it can be assumed that plasma concentration of ketamine in our animals would have reached beyond that reported previously in dogs [39].

In this study, KLD and LLD bolus dose significantly reduced MF at 5 minutes after treatment. However, the bolus was not effective in suppressing MF at 20 and 40 minutes posttreatment stimulus. It was noted that MLX (0.2 mg/kg IV) did not suppress the rise in MF among the experimental animals. In fact, similar effects of the specific COX-2 inhibitor (Parecoxib) have been reported by Kongara et al. [15] and Peng et al. [45]. In fact, Kongara et al. [15] also reported that parecoxib did not reduce MF after electrical stimulation in dogs [15]. But Peng et al. [45] reported that parecoxib did not produce significant effects on neural activity in response to mechanical stimulation in rats [45]. Similar effects of meloxicam have been reported by Santos et al. [46] where meloxicam did not show significant effect on the hot plate test in mice [46]. In another study, specific COX-2 inhibitor (parecoxib) inhibited behavioral changes in carrageenan induced inflammation but had no effects on acute pain models such as acetic acid induced writhing and formalin test [47]. Surgical injury produces inflammation and upregulation of COX-2 in the injured tissue, while acute electrical stimulus (used in this study) activates A*β*, A*δ*, and C fibers without expression of abnormal COX-2. Absence of peripheral inflammation as well as abnormal expression of COX-2 might be the reason why meloxicam did not show its effect in this study.

Poststimulation changes in Ptot were variable between the treatments. Relationship between *E*_THAL_ and Ptot in response to surgical castration in horses has been reported by Murrell et al. [48]. It was suggested that the decrease in the Ptot indicates reduction in the adequacy of anaesthesia due to noxious stimuli. The variation in the decrease of Ptot may be due to differences in the *E*_THAL_ of the individual animal. Morphine prevented changes in Ptot at 5, 20, and 40 minute stimulation in this study. Conversely, in the previous study by Kongara et al. [15], Ptot is not changed among animals that were treated with morphine [15]. It should be noted that Ptot did not change at 20 and 40 minutes posttreatment stimulation in the LLD group. In contrast to MRP, LLD, and LCRI groups, KCRI had no effect on Ptot values as reflected in MF. Changes in Ptot and power in various frequency bands correlated closely with the transition from consciousness to unconsciousness during induction of anaesthesia in humans, thus reflecting that EEG power is associated with the depth and the adequacy of anaesthesia [49–51].

Electrical stimulation is a noninvasive, quantifiable, and reproducible nociceptive stimulus. Furthermore, it also provides synchronized afferent signals [22]. Electrical stimulation produced greater action potential and most consistent EEG responses compared to mechanical and thermal stimulation, [52] resulting in a significant increase in MF [15]. Noxious electrical stimuli can be used to evaluate the efficacy of centrally acting agents. This type of stimulation activates all peripheral afferent fibers (A*β*, A*δ*, and C fibers) nonselectively and bypasses the transduction mechanism. This mechanism, however, can be advantageous in studies using drugs administered systematically to evaluate their effects on the central nervous system (CNS), provided that the drug has no effect on peripheral fibers [22]. The results of this study further reaffirmed the practicality of using noxious electrical stimuli to evoke consistent EEG response when evaluating potential antinociceptive potentials of pharmacologic agents. The current study used a peripheral nerve stimulator N272 (Fisher and Paykel Healthcare international, New Zealand) with an electrical stimulation of 40 mA at 50 Hz frequency for 5 seconds. This particular amperage of 40 mA was reported to be able to elicit supramaximal stimulus in human subjects [42].

Detection of the perception of noxious stimuli in the brain typically begins with arousal or desynchronization. Desynchronization is a typical EEG response characteristic of nociception [14, 15, 19, 20, 45, 48, 52]. Arousal or “desynchronisation” is the shifting of EEG from high amplitude, low-frequency waves (commonly seen in anaesthesia) to low-amplitude, high-frequency waves (resembling that of awakeness) in response to noxious stimulus [12]. Previous study in the dogs also reports desynchronization in response to electrical stimulation [15]. Meloxicam did not prevent the occurrence of desynchronization compared to morphine, ketamine, and lidocaine. Antinociceptive effects of meloxicam after systemic injection are mainly due to its action in the periphery or near the nociceptor endings [53]. Nonsteroidal anti-inflammatory drugs (NSAIDs) have been reported to produce analgesia through activation of descending modulatory systems, inhibiting the excitation of the spinal dorsal horn neurons, in the absence of peripheral inflammation [54]. It was possible that meloxicam was unable to prevent the flow of nociceptive action potentials to the cerebral cortex following the acute electric stimulus. This concurred with the findings from previous study [55] that various types of analgesics can be classified by their effects on the EEG spectrum. Changes in EEG spectrum have been used as a tool to evaluate antinociceptive effects of drugs [13, 14].

Heart rates increased transiently at pretreatment stimulation in all treatment groups. Similarly, the heart rates were also increased at posttreatment stimulation; however, this posttreatment stimulation increase was smaller than that of pretreatment stimulation. Similar results have been reported in piglets during castration, where heart rate and blood pressure increase were smaller after intratesticular lidocaine injection than those in control. Noxious stimulation can cause changes in sympathetically mediated cardiovascular parameters in animals under general anaesthesia [17, 56]. A rise in heart rate and blood pressure has been reported to be associated with the increase or decrease in various frequency bands of the EEG spectrum in response to nociceptive stimulus in sheep and dogs [17, 57]. Haga and Dolvik observed no significant change in EEG variables in relation to a significant rise in mean arterial pressure in horses under castration [58]. In this study, posttreatment heart rates increased in response to nociceptive stimulation, which were transient and time-linked to noxious stimulus.

The mechanism by which systemic lidocaine exerts its antinociceptive effect is not yet fully understood [59]. Numerous effects of systemic lidocaine have been reported that cannot be explained by its main mechanism of action (action on voltage gated sodium channels). In addition to exerting its effect on voltage gated sodium channels [60], lidocaine inhibited the release of glutamate from cerebrocortical nerve terminals [61] and increased extracellular glycine concentration resulting in enhanced activity at inhibitory glycinergic synapses by inhibition of GlyT1-mediated glycine reuptake [62]. Upregulation of the sodium channels at the site of injury partly produced peripheral hyperexcitability [63], which, in turn, is responsible for the nociceptive perception felt. Tanelian & MacIver [64] described that lidocaine specifically blocks peripheral ectopic discharges in neurons involved in nociception [64]. Lidocaine was reported to be able to reduce postoperative hyperalgesia by acting on Na^+^ channels [65]. Various mechanisms of actions, including peripheral and central sites of action, have been discussed for its antinociceptive action [66]. Thus, intravenous lidocaine produces analgesia as a result of its multifactorial interaction with Na^+^ channels and direct or indirect interaction with different receptors and nociceptive transmission pathways [67]. Ketamine, on the other hand, blocks the calcium channels at the NMDA receptors resulting in the inhibition of the nociceptive action potentials from being transmitted upward, thus blocking the pain sensation [68]. Location of NMDA-glutamate receptors on peripheral nerves has been described in animal studies [69]. Other proposed actions of ketamine include its action on muscarinic [70], nicotinic [71], and the *δ*, *μ*, and *κ*-opioid receptors [72]. It blocks Na^+^ channels in peripheral and central nervous system [71] and interacts with monoaminergic and voltage-sensitive Ca^+2^ channels [73]. Systemic lidocaine did not diminish or abolish the brain response to innocuous or acute noxious electric stimulation based on functional magnetic resonance imaging, blood-oxygen-level-dependent (fMRI-BOLD) conducted on rats [74]. However, studies have found that morphine suppressed the brain's response to acute noxious electrical stimulation [75]. Crisp et al. reported that ketamine-induced analgesia, measured by increased tail flick latency in rats, was reversed by serotonin antagonist (methysergide), alpha adrenoceptor agonist (phentolamine), and naloxone [76]. However, tail flick latency did not increase when spinal ketamine was administered to rats with bilateral dorsal funiculus (DLF) lesions in a system where morphine was effective. Higher doses of naloxone were required to reverse the spinal action of ketamine than needed to block the effect of morphine. This suggests descending inhibitory monoaminergic pain pathways as well as different, less sensitive opiate receptor subtype [77] involved in the ketamine's analgesic action. But morphine's analgesic action in DLF lesioned rats was due to its action on intrinsic *μ* opiate receptors in the dorsal horn [78]. These lines of evidences suggest the different pathways involved in the analgesic effects of morphine, lidocaine, and ketamine. In this study, morphine, ketamine, and lidocaine significantly attenuated the MF, suggesting an antinociceptive effect through multiple pathways.

## 5. Conclusions

In conclusion, this study demonstrated that ketamine, lidocaine, and morphine at the dosage of 10 mcg/kg/min, 50 mcg/kg/min, and 0.2 mg/kg, (IV), respectively, demonstrated evident MF suppression. On the contrary, meloxicam failed to prevent an increase in MF. That might be due to the inability of meloxicam to inhibit transmission of afferent nociceptive stimuli to the cerebral cortex in response to acute electrical stimulation. Changes in Ptot seem to be not directly related to nociception as in MF. Thus, ketamine and lidocaine produced antinociception in response to acute nociceptive electrical stimulus.

## Figures and Tables

**Table 1:** Pre- and posttreatment absolute values (mean ± SE) of MF (Hz) with their percentage change (mean values) after lidocaine and ketamine infusion.

Treatment	Before treatment	% change	Dose 1	% change	Dose 2	% change
MF (LCRI)						
Before stimulation	8.88 ± 2.58^c^		8.75 ± 2.01^c^		9.03 ± 1.38^c^	
After stimulation	16.82 ± 4.44^a^	118.84	13.52 ± 2.38^b^	94.88	13.5 ± 3.06^b^	66.93
MF (KCRI)						
Before stimulation	9.78 ± 1.54^c^		10.54 ± 3.84^c^		8.72 ± 3.1^c^	
After stimulation	20.21 ± 3.59^a^	104.84	13.33 ± 3.71^b^	57.69	12.69 ± 3.68^b^	55.08

Means within a row and column in each treatment group followed by different letters are significantly different at (*P* < 0.05). MF = median frequency. LCRI = lidocaine continuous rate infusion. Dose 1 = 2 mg/kg loading + 50 mg/kg/min (lidocaine) and 3 mg/kg loading + 10 mg/kg/min (ketamine). Dose 2 = 100 mg/kg/min (lidocaine) and 50 mg/kg/min (ketamine).

**Table 2:** Pre- and posttreatment absolute values (mean ± SE) of MF (Hz) with their percentage change (mean values) at various stimulation times across treatment groups.

Treatment	Before treatment	% change	5 min	% change	20 min	% change	40 min	% change
MF (KLD)								
Before stimulation	12.17 ± 4.57^b^		11.26 ± 7.65^b^		15.64 ± 11.04^ba^		11.53 ± 3.28^b^	
After stimulation	20.1 ± 4.15^a^	108.18	11.03 ± 7.27^b^	25.07	19.02 ± 9.76^a^	88.12	16.13 ± 3.49^ba^	63.38
MF (LLD)								
Before stimulation	11.93 ± 2.55^bc^		7.76 ± 1.05^c^		11.81 ± 2.88^bc^		7.97 ± 0.86^c^	
After stimulation	24.11 ± 6.58^a^	112.17	13.19 ± 2.58^b^	65.52	21.74 ± 5.44^a^	83.87	21.06 ± 5.74^a^	155.19
MF (MRP)								
Before stimulation	9.09 ± 1.47^c^		9.29 ± 2.53^c^		9.14 ± 2.47^c^		9.87 ± 2.53^c^	
After stimulation	20.58 ± 1.57^a^	172.69	15.39 ± 4.34^b^	104.41	14.94 ± 1.93^b^	69.78	13.46 ± 1.24^b^	72.05
MF (MLX)								
Before stimulation	6.23 ± 0.56^c^		8.05 ± 0.77^bc^		9.42 ± 1.09^b^		9.7 ± 1.46^b^	
After stimulation	14.4 ± 3.64^a^	124.42	16.99 ± 2.76^a^	121.3	16.79 ± 3.65^a^	82.8	17.11 ± 2.8^a^	75.25

Means within a row and column in each treatment group followed by different letters are significantly different at (*P* < 0.05). MF = median frequency. KLD = ketamine loading dose. LLD = lidocaine loading dose. MRP = morphine. MLX = meloxicam.

**Table 3:** Pre- and posttreatment absolute values (mean ± SE) of Ptot (*µ*V^2^) with their percentage change (mean values) after continuous lidocaine and ketamine infusion.

Treatment	Before treatment	% change	Dose 1	% change	Dose 2	% change
Ptot (LCRI)						
Before stimulation	8.51 ± 1.14^a^		7.87 ± 0.97^a^		8.75 ± 1.04^a^	
After stimulation	8.04 ± 1.42^a^	10.41	6.32 ± 0.75^b^	18.87	6.48 ± 0.78^b^	25.46
Ptot (KCRI)						
Before stimulation	8.44 ± 1.3^a^		9.24 ± 0.97^a^		8.63 ± 1.18^a^	
After stimulation	7.25 ± 1.08^b^	14.13	6.82 ± 1.11^b^	27.41	6.52 ± 0.9^b^	21.85

Means within a row and column in each treatment group followed by different letters are significantly different at (*P* < 0.05). Ptot = total power of the EEG. LCRI = lidocaine continuous rate infusion. Dose 1 = 2 mg/kg loading + 50 mg/kg/min (lidocaine) and 3 mg/kg loading + 10 mg/kg/min (ketamine). Dose 2 = 100 mg/kg/min (lidocaine) and 50 mg/kg/min (ketamine).

**Table 4:** Pre- and posttreatment absolute values (mean ± SE) Ptot (*µ*V^2^) with their percentage change (mean values) at various stimulation times across treatment groups.

Treatment	Before treatment	% change	5 min	% change	20 min	% change	40 min	% change
Ptot (KLD)								
Before stimulation	8.17 ± 1.08^c^		10.71 ± 0.84^a^		10.41 ± 1.42^a^		9.18 ± 1.22^bc^	
After stimulation	6.44 ± 0.52^d^	17.54	10.02 ± 1.22^ab^	12.42	8.07 ± 1.79^c^	25.11	6.76 ± 0.9^d^	26.96
Ptot (LLD)								
Before stimulation	9.01 ± 0.94^a^		8.17 ± 0.73^ab^		8.52 ± 0.68^ab^		7.8 ± 0.76^cb^	
After stimulation	8.29 ± 0.85^ab^	19.8	6.69 ± 0.85^d^	19.04	8.1 ± 1.35^ab^	37.04	7.1 ± 0.83^dc^	23.6
Ptot (MRP)								
Before stimulation	7.78 ± 0.65^ab^		8.51 ± 1.11^a^		7.86 ± 0.66^ab^		7.77 ± 0.73^ab^	
After stimulation	6.29 ± 0.63^c^	7.06	8.49 ± 1.01^a^	14.92	7.04 ± 0.89^bc^	17.36	7.02 ± 0.59^bc^	16.32
Ptot (MLX)								
Before stimulation	9.04 ± 1.32^a^		9.28 ± 1.1^a^		8.57 ± 1.15^ab^		8.19 ± 0.92^ab^	
After stimulation	7.86 ± 1.52^bc^	17.22	7.08 ± 0.76^c^	23.04	6.88 ± 0.67^c^	22.02	7.04 ± 0.87^c^	14.27

Means within a row and column in each treatment group followed by different letters are significantly different at (*P* < 0.05). MF = median frequency. KLD = ketamine loading dose. LLD = lidocaine loading dose. MRP = morphine. MLX = meloxicam.
